# Recent progress and future perspectives of infrared light-based technology in ophthalmology

**DOI:** 10.3389/fmed.2026.1888199

**Published:** 2026-07-10

**Authors:** Meihua Ding, Wei Sun, Tailiang Lu, Yuanyuan Hu, Fang Sha, Kai Tang, Shihai Miao, Hongsheng Bi

**Affiliations:** 1 Affiliated Eye Hospital of Shandong University of Traditional Chinese Medicine, Jinan, China; 2 Shandong Academy of Eye Disease Prevention and Therapy, Shandong Provincial Clinical Medical Research Center of Optometry and Children Visual Impairment Prevention and Control, Jinan, China; 3 Ophthalmology and Optometry Medical School, Shandong University of Traditional Chinese Medicine, Jinan, China; 4 Shandong Non-Metallic Materials Institute, Jinan, China

**Keywords:** eye movement, IR imaging, IR light-based technology, ocular surface temperature, ophthalmology

## Abstract

Infrared (IR) light provides non-invasive, depth-resolved imaging and diagnostic capabilities, allowing for precise tissue analysis and real-time monitoring across various applications. In recent years, IR light-based technologies—encompassing IR spectroscopy, IR imaging, IR thermography, IR eye tracking, and IR therapy—emerged as a powerful tool in ophthalmology, offering valuable insights into multiple aspects of eye health. Its ability to detect subtle changes in ocular tissues and deliver real-time data holds significant potential to enhance both diagnostic accuracy and treatment efficacy in eye care. This review highlights recent advances in the application of IR light-based technology in ophthalmology, focusing on applications such as quantitative ocular surface thermography, depth-resolved structural imaging, functional monitoring, and IR based therapeutic applications in ophthalmology. IR light’s ability to penetrate ocular tissues non-invasively allows for detailed assessments of eye physiology, facilitating early disease detection and supporting personalized treatment strategies. While IR technology exhibits great potential, key challenges remain in its optimization—notably improving spectral resolution, depth penetration, and device miniaturization for expanded clinical integration. Finally, the current challenges and future prospects of IR light based-technology in ophthalmology are discussed, contributing to early diagnosis, monitoring disease progression, and evaluating treatment outcomes.

## 1 Introduction

IR light was the first non-visible region of the spectrum to be discovered and systematically investigated (1–3). It was initially identified by William Herschel, who also demonstrated its particularly strong thermal properties (4). In 1800, Herschel used a prism to disperse sunlight and placed thermometers at different wavelengths and color bands to accurately measure the heating effect produced by each color. Notably, the thermometer located beyond the red region recorded the most significant temperature increase. IR light-based technology has developed into a rapid, convenient, non-invasive, and non-destructive analytical technique, offering a range of exceptional spectral properties (5–8). These include the ability to detect phenomena not visible to the naked eye, significant light scattering effects, deep penetration capability, low absorption of incident light, and minimal scattering and absorption within biological tissues (9–11). Therefore, IR light-based technology has found successful applications across various fields, including petrochemical engineering, astronomical research, fiber optic communications, biomedical sciences, security surveillance, and food inspection (12–14).

Since the invention of the incandescent lamp in the 19th century, researchers have developed numerous IR emitters (15–19). In 1961, the first narrow band IR light emitting diode (*λ*_em_ = 900 nm) was fabricated using a gallium arsenide (GaAs) substrate by Texas Instruments (20, 21). IR emitters based on InGaAs semiconductors provide benefits including high radiant power, long lifetime, and compact dimensions (22–24). Inspiringly, in 2016, taking advantage of the mature commercialization of white light LEDs based the highly efficient InGaN blue light chips (25), Germany’s Osram developed the first blue-light-excited IR light-emitting material conversion device (SFH 4735) (26). The continuous advancement of IR devices has driven the widespread application of IR light based technology, particularly in the medical field (27).

The potential of IR light-based technology for medical applications was first revealed in 1977 (28), when Jöbsis investigated the hemoglobin oxygenation and deoxygenation states in cats. With the advantages of non-invasiveness and continuous monitoring, this technology has been extensively utilized across a range of fields, including epilepsy (29), diabetes (30), cancer (31), neurological disorders (32, 33), and other physiological conditions (34–36). For example, Qiao et al. reported the IR light-mediated PadC optical switching system for cancer therapy (31). When illuminated by 710 nm light, the chimeric photoreceptor PadC converts intracellular guanosine triphosphate into cyclic di-guanosine monophosphate. This process activates the transcription factor MrkH, enabling it to bind to the chimeric promoter PmrkA and initiate the expression of target genes. Notably, the system exhibits a 55-fold increase in reporter gene expression under IR illumination compared to dark conditions. Meanwhile, several researchers have reviewed the applications of IR light-based technology in medical diagnostics, pharmaceutical analysis, imaging, and phototherapy (37–42). With ongoing advancements in IR light-based technology, ophthalmology has progressively emerged as a significant field of application (43, 44). As a light sensitive organ, the eye exhibits high responsiveness to external light (45). Due to the deeper penetration of IR light relative to visible light and its favorable tissue safety profile on ocular tissues, IR light-based technology has become an ideal tool for both clinical research and treatment in ophthalmology (46, 47). IR light-based technology in ophthalmology encompass IR spectroscopy, IR imaging, IR thermography, IR eye tracking, and IR therapy (48). Table 1 summarizes the definitions and distinctions of these technologies. Thanks to IR thermal imaging, monitoring the surface temperature of the eye has become an effective method for detecting a variety of ocular conditions, including dry eye, retinal disorders, and glaucoma (43, 48). The unique penetrating properties of IR light allow for the imaging of both the ocular surface and internal structures, offering a comprehensive view of eye health (46, 49). Additionally, the non-contact nature of IR technology enables real-time tracking of eye movement (50). Furthermore, IR light-based therapies such as phototherapy, photothermal therapy, and photodynamic therapy have proven to be valuable tools in the treatment of various ocular diseases, leveraging the therapeutic potential of light to address complex conditions (44, 51).

**Table 1:** Definitions and distinctions of IR light-based technology in ophthalmology.

Technology	Definition	Applications
IR spectroscopy	Measurement of absorption, reflection, or emission spectra of IR light by tissues	Tissue composition analysis, oxygen saturation monitoring
IR imaging	Capture of reflected IR light to visualize tissue structures	Fundus imaging, meibography, lacquer crack detection
IR thermography	Detection of thermal infrared radiation emitted by tissues	Temperature measurement, dry eye diagnosis, glaucoma screening
IR eye tracking	Real-time monitoring of eye position and movement using IR illumination	Vestibular function assessment, sleep monitoring, facial recognition
IR therapy	Therapeutic application of IR laser/light to induce biological effects	Diabetic macular edema treatment, meibomian gland dysfunction, myopia

The current developments in IR light-based technology in the field of ophthalmology are systematically summarized in this review. Specifically, we examine the fundamental principles and technological innovations of IR light-based technology, with a particular focus on its applications in thermography, imaging, eye movement monitoring, and the treatment of ocular diseases. This review gives a comprehensive overview of key applied research, highlighting the advantages of IR light-based technologies over traditional optical techniques, including favorable penetration depth, enhanced sensitivity, and non-invasive capabilities. Finally, we address the challenges and limitations hindering the widespread clinical adoption of IR light-based technology and propose potential directions for future development, offering insights to support the continued progress of IR light-based technology in both ocular research and clinical practice.

## 2 Search strategy and selection criteria

A comprehensive literature search was conducted across the following electronic databases: PubMed/MEDLINE, Web of Science, and Google Scholar. No restrictions were placed on publication date to ensure comprehensive coverage of both historical milestones and recent advances. The following search terms and combinations were used: (“infrared” OR “IR”) AND (“ophthalmology” OR “eye” OR “ocular”). Only English language publications were included.

## 3 IR thermography in ophthalmology

Temperature is crucial for maintaining ocular tissue health and function, making its monitoring vital in the diagnosis and management of various ocular disorders (52–54). Abnormal thermal changes may signal underlying pathologies, including inflammation, infection, and retinal diseases. Various methods have been employed to measure ocular surface temperature (OST), including thermocouples and temperature-sensitive resistors (48). However, a major drawback of these techniques is the need for direct contact with the ocular surface, which is often uncomfortable and undesirable for patients (55, 56). Additionally, the area of contact can introduce variability in the measurement results (57). IR light-based technology enables non-invasive assessment of tissue properties and has emerged as a promising tool for ocular temperature monitoring. Table 2 summaries the key parameters of recently reported IR thermography in ophthalmology.

**Table 2:** Key parameters of recent reported IR thermography in ophthalmology.

Disease	IR light	Sample size	Diagnostic performance and limitation	References
Dry eye	8–13 μm	Dry eye group: 36, control group: 27	OST, radial temperature difference. Emissivity; costly equipment	Morgan et al. (63)
Dry eye	7–18 μm	Dry eye group: 51, control group: 51	OST, OST difference (open/close eye). Accuracy, limited surface temperature	Singh et al. (55)
Dry eye	7–14 μm	Dry eye group: 82,control group: 26	OST decay analysis. Accuracy, algorithm simplification	Chiang et al. (56)
Retinopathy (NPDR)	8–14 μm	NPDR group: 51, control group: 53	OST. Cannot quantitative measure blood flow	Sodi et al. (60)
Glaucoma	8–14 μm	Glaucoma group: 45, control group: 20	OST. Ignore temperature caused by inflammation and medication	Zadorozhnyy et al. (61)
Glaucoma	17 μm	Glaucoma group: 21, control group: 19	OST, cooling rate. Limited sample size, temperature changes caused by medication	García-Porta et al. (53)
Keratoconus	8–14 μm	Keratoconus group: 10, control group: 17	OST, OST difference (6 s). Limited sample size, accuracy	Gu et al. (57)

### 3.1 OST in dry eye

**Figure 1:**
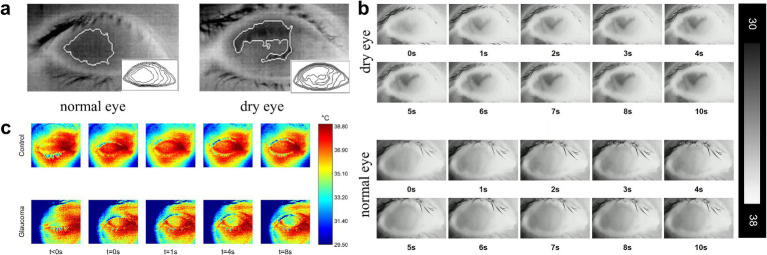
**(a)** The contour of the relative-lower-temperature-area and the equal temperature boundary of the OST of normal eye and dry eye. Reproduced from Tai Yuan et al. (62) under the CC BY 4.0 license. **(b)** IR thermal images of the normal eye and dry eye over 10 s following the start of measurement, with a temperature scale from 30 to 38 °C. Reproduced from Ref. Wu et al. (59) under the CC BY 4.0 license. **(c)** Thermal images of both glaucoma and healthy eyes were captured before eye opening, and at 1, 4, and 8 s after opening. Reproduced from García-Porta et al. (53) under the CC BY 4.0 license.

Notably, IR thermometry of the ocular surface has been established as a method for detecting and diagnosing dry eye. The National Eye Institute/Industry Workshop defines dry eye disease as a disorder of the tear film due to tear deficiency or excessive tear evaporation, which can cause ocular discomfort, decrease visual acuity, and lead to a decline in visual function and quality of life (57–63). The pioneering work of Morgan et al. in 1995 marked the use of IR thermography to assess the tear film in patients with dry eye syndrome (63). Subsequently, in 2011, Chiang’s group developed a non-contact IR thermal imaging system to measure the spatial and temporal variation of OST (Figure 1a) (62). The compactness value for the relatively low-temperature area was defined as perimeter^2^/area, and the compactness value were calculated to be 16 and 59 for normal eye and dry eye, respectively. Furthermore, insets display the corresponding isothermal boundaries, revealing that the boundary in normal eyes is smooth and roundish, whereas in dry eyes it is more irregular. In 2016, Tan et al. reported that IR thermography technology demonstrates excellent reproducibility in assessing 10 indicators of OST, making it a valuable tool for research on dry eye disease (58). Recently, Wu et al. studied the corneal temperature in normal eye and dry eye through a high-resolution IR thermography (59). The IR thermal camera features a frame rate of 30 Hz, a spatial resolution of 640 × 512 pixels (pixel size: 14 × 14 μm), and a measurement accuracy of ±2%. Figure 1b shows the IR thermal images of the normal eye and dry eye over 10 s following the start of measurement, with a temperature scale from 30 to 38 °C. In contrast to the stable gray level and temperature observed in normal eye over the 10 s, the OST of dry eye exhibits a gradual decline trend, corresponding to a shift from high to low gray levels in the thermal images.

Current clinical diagnosis of dry eye relies on the Schirmer test, tear break-up time (TBUT), and ocular surface staining, which require topical direct contact (46, 55). IR thermography provides non-contact, real time evaluation of tear film dynamics (58, 62). However, its diagnostic performance against these standard tests has not been validated in large cohorts (58). Therefore, thermography remains a complementary research tool rather than a replacement for routine clinical testing.

### 3.2 OST in retinal diseases and glaucoma

In addition to diagnosing dry eye, IR thermography can also be used to detect ocular diseases including diabetic retinopathy and glaucoma. In 2009, Sodi et al. applied IR thermography to monitor diabetic retinopathy and observed a significant decrease of OST in patients, which was attributed to reduced ocular blood flow (60). In 2018, Zadorozhnyy et al. employed IR thermography to examine OST variations before and after transscleral cyclophotocoagulation in patients with absolute-stage glaucoma (61). The OST of the affected eye was significantly lower compared to that of the healthy contralateral eye. As intraocular pressure decreased, the temperature of the treated eye gradually increased, eventually reaching levels comparable to those of the healthy eye. Porta et al. reported the dynamic changes of OST during the moment of eye opening in glaucoma patients and healthy individuals (53). As shown in Figure 1c, the glaucoma patient exhibited lower OST and faster cooling rates ascribed to insufficient ocular blood flow and tear film instability. Overall, the application of IR thermometry technology in OST detection provides a theoretical basis for the early screening of ocular diseases such as dry eyes, diabetic retinopathy and glaucoma.

For diabetic retinopathy and glaucoma, standard assessments include fundus photography, optical coherence tomography (OCT), fundus fluorescein angiography (FFA) and visual field testing (48, 60). Ocular surface temperature changes in these diseases reflect altered ocular perfusion (60, 61). Although IR thermography is non-invasive and rapid, current evidence is limited to small pilot studies (53, 60, 61). Prospective trials comparing its diagnostic accuracy with established methods are needed before clinical adoption.

## 4 IR imaging in ophthalmology

IR imaging is a noninvasive, nonionizing, and real-time modality that has emerged as a valuable diagnostic tool for various *in-vivo* and *in-vitro* applications (64–66). This technique provides a straightforward and highly sensitive approach for ophthalmology imaging research (67–69).

### 4.1 IR meibography for dry eye

**Figure 2:**
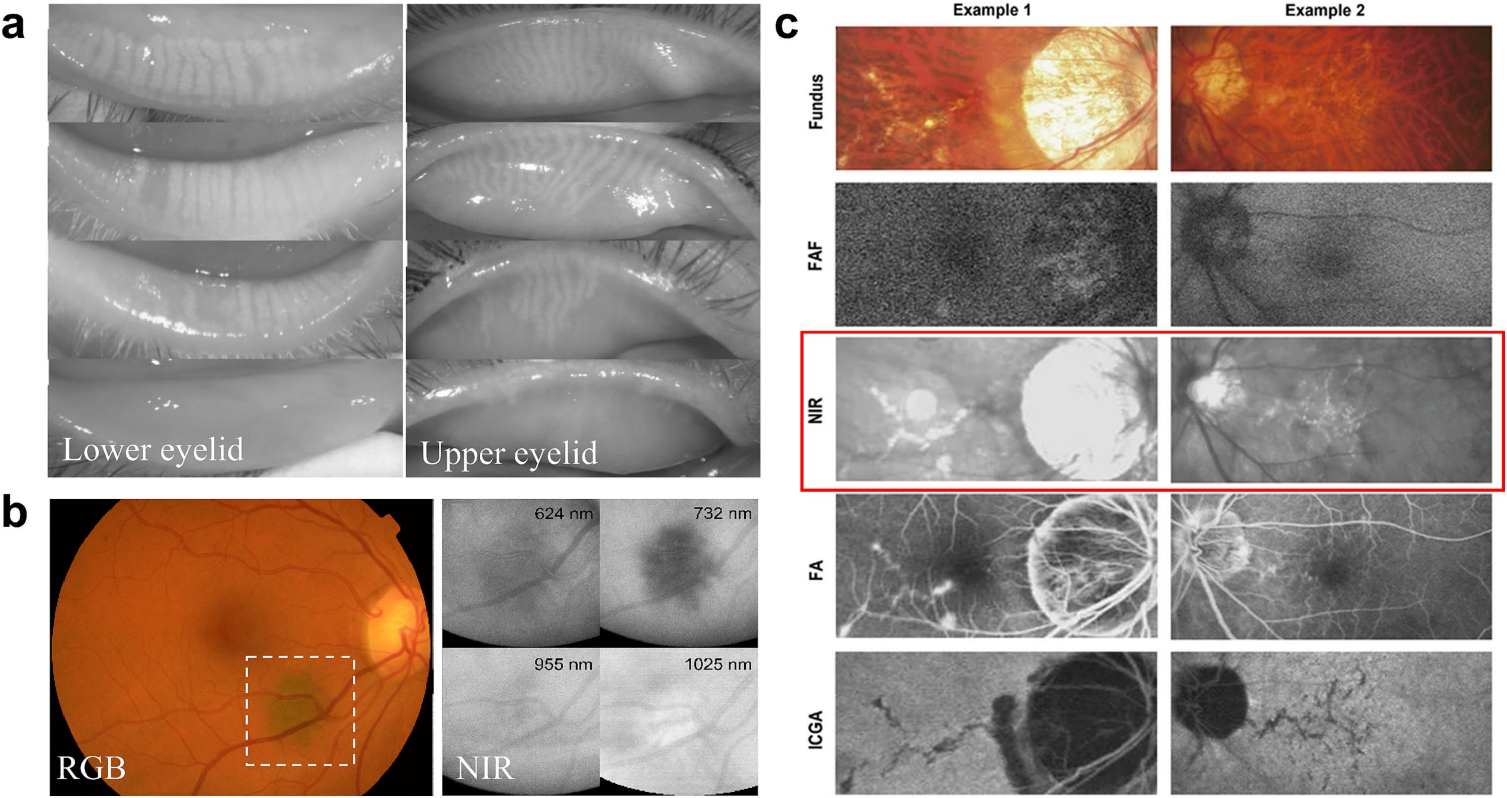
**(a)** IR imaging of the lower and upper eyelids with different grades of meibomian gland dysfunction. Reproduced with permission from Finis et al. (76). Copyright (2015) Taylor & Francis. **(b)** RGB fundus image and IR imaging of the eye fundus with a choroidal nevus. Reproduced from Burgos-Fernández et al. (84) under the CC BY 4.0 license. **(c)** Multimodal imaging of the eyes fundus with lacquer cracks. Reproduced with permission from Liu et al. (85). Copyright (2014) Springer Nature.

In the field of ocular surface health, IR imaging is widely utilized for diagnosing dry eye disease (70). Specifically, IR meibography serves as a direct clinical method for visualizing the meibomian glands in two dimensions (71, 72). It allows clear identification of structural abnormalities, such as meibomian glands atrophy and drop-out, thereby confirming pathological changes in the meibomian glands (73). In 1982, Jester et al. first employed IR imaging of the meibomian glands to observe glandular structures and identify early structural changes in the meibomian glands (74). And then, Srinivasan et al. achieved non-contact imaging of the meibomian glands using Keratograph 4 device and investigated its applications in diagnosing meibomian gland dysfunction and dry eye symptoms (75). In 2014, Finis et al. employed IR meibography to investigate the relationship between meibomian gland atrophy and glandular function (76). Figure 2a displays the IR imaging of the lower and upper eyelids with different grades of meibomian gland dysfunction. The grading of meibography from top to bottom is recognized as 0 point (no meibomian gland defect), 1 point (less than one-third meibomian gland defect), 2 points (less than two-thirds meibomian gland defect), and 3 points (more than two-thirds meibomian gland defect), respectively. Recently, Persiya et al. reviewed the application of IR thermography combined with deep learning in diagnosing dry eye syndrome, concluding that this approach represents the future direction for non-invasive automated diagnosis of the condition (77). However, larger-scale and standardized data sets are still required to support its implementation. Table 3 summaries the key parameters of recently reported IR meibography for dry eye.

**Table 3:** Key parameters of recently reported IR meibography for dry eye.

IR light	Sample size	Limitation	References
880 nm	37 subjects	Requires lid eversion, high equipment cost	Srinivasan et al. (75)
8–14 μm	81 subjects	Small sample size, requires dedicated thermal camera	Acharya et al. (70)
8–14 μm	83 dry eye + 21 normal	Age mismatch between groups, small sample	Acharya et al. (64)
880 nm	11 dry eye + 10 controls	Small sample, no non-SS dry eye control, disease duration uncontrolled	Menzies et al. (66)
8–14 μm	62 dry eye + 63 controls	Low specificity (~50–62%), only mild-to-moderate dry eye	Tan et al. (73)
1,310 nm	115 eyes/64 patients	Small FOV (4.3 × 4.3 mm), upper lid only	Yoo et al. (72)
≥ 720 nm	99 eyes/91 patients	Single center, subjective pressure control; sample size not pre-calculated	Ha et al. (67)

Slit-lamp examination primarily assesses meibomian gland orifices and meibum expressibility but provides no information on internal glandular structure. *In vivo* confocal microscopy provides meibomian gland details but is time consuming and unsuitable for routine screening. IR meibography directly visualizes glandular structure and atrophy (76), and devices such as Keratograph have been integrated into clinical practice (75). Subjective grading remains a limitation and deep learning approaches may improve objectivity but require multi center validation (76, 77). Compared with invasive histopathology, IR meibography represents a practical non-invasive tool for morphological assessment of meibomian glands.

### 4.2 IR fundus imaging for retinal and choroidal disease

More importantly, compared to visible light, IR light can penetrate the eye’s transparent structures and offers more effective penetration of deeper tissues at the back of the eye, including the retina and choroid (78–81). 820 nm IR light reveals the microstructure of retinal pigment epithelial (RPE) cells, 895 nm light visualizes subretinal structures, such as drusen and Bruch’s membrane thickening, and 960–1,300 nm light enables the evaluation of deeper RPE and choroidal structures, respectively (80–82). Therefore, these properties grant IR imaging a unique advantage in the detection of retinal pathologies (82, 83). Burgos-Fernández et al. employed multispectral fundus imaging to enhance early diagnostic capabilities for retinal and choroidal diseases (Figure 2b) (84). The nevus exhibits good contrast within the red-IR spectrum (600–1,000 nm). The dark-to-light contrast transition observed in pigmented nevi between 955 and 1,025 nm may reflect their etiological status, intrinsic condition, and the state of the adjacent retinal pigment epithelium. Consequently, spectral information could aid in differentiating benign from malignant lesions by providing enhanced data on pigmentation status. Liu et al. evaluated the diagnostic performance of multimodal imaging in detecting lacquer cracks in eyes with high myopia caused by age-related macular degeneration (AMD) (85). The multimodal imaging of the eyes fundus with lacquer cracks are shown in Figure 2c. Fundus photographs reveal lacquer-crack-like lesions presenting as yellow linear and punctate patterns. Fluorescein angiography (FA) fails to fully delineate the extent and morphology of lacquer-crack lesions. Notably, IR imaging reveals highly reflective lines that correspond to the low-fluorescence lines seen in late-phase indocyanine green angiography (ICGA). Additionally, IR imaging technology enables the monitoring of eye fatigue and blood drug concentrations, as well as the detection of cataracts (86–90) and other anterior segment disorders (91). Table 4 summaries the key parameters of recently reported IR fundus imaging for retinal and choroidal disease.

**Table 4:** Key parameters of recently reported IR fundus imaging for retinal and choroidal disease.

Disease	IR light	Sample size	Limitation	References
Macular drusen, cataract	830 nm	/	Image clarity, image quality affected by media opacity; no quantitative performance metrics	Manivannan et al. (78)
AMD	795–915 nm	/	No quantitative performance data	Elsner et al. (82)
AMD	795–895 nm	50 controls; >100 patients	8-bit digitization limits dynamic range; axial resolution limited by pinhole size	Elsner et al. (81)
Optic disc pit maculopathy	820 nm	3 patients	Very small sample, non-comparative	Hiraoka et al. (68)
Choroidal nodules	815 nm	95 patients; 100 controls	Single-center, Caucasian population, affected by refractive error and iris color	Viola et al. (79)
High myopia – lacquer cracks	787 nm	34 patients	Retrospective, no quantitative performance metrics	Liu et al. (85)
Acute zonal occult outer retinopathy	813 nm	10 patients	Small sample, hyperreflectance only visible near borders of abnormal ISe band	Ueno et al. (80)
Geographic atrophy	/	97 patients	Cannot identify junctional features	Abdelfattah et al. (69)
AMD	795 nm	15 patients; 7 controls	Challenging in elderly patients, RPE mosaic not always resolvable	Vienola et al. (83)
AMD, glaucoma, choroidal nevus	400–1,300 nm	30 patients; 137 controls	Prototype stage, limited FOV, restricted by refractive error and astigmatism	Burgos-Fernández et al. (84)

FFA and ICGA are considered clinical standards for detecting vascular leakage, neovascularization, and retinal/choroidal circulation. However, they require intravenous contrast injection and may cause adverse reactions. IR imaging is non-invasive, contrast free, and offers superior visualization of pigmented lesions (choroidal nevi) and lacquer cracks (84, 85). For these specific indications, IR imaging serves as a complementary adjunct to FFA, ICGA, and OCT within a multimodal strategy (85).

## 5 Functional monitoring: IR eye tracking and pupillometry

Eye-tracking technology is widely used in the fields of neuroscience, psychology, medical diagnostics, virtual reality, and human-computer interaction (92–95). Traditional methods for monitoring eye movements—such as electrooculography (EOG), search-coil technique, and scleral contact lenses—are limited by invasiveness, low accuracy, poor comfort, and restricted environmental adaptability, thereby hindering their practical application (96–98). Thanks to the advantages of detection accuracy, real-time performance, and non-invasive, IR light has become a focus of research in eye-tracking application (99–101).

**Figure 3:**
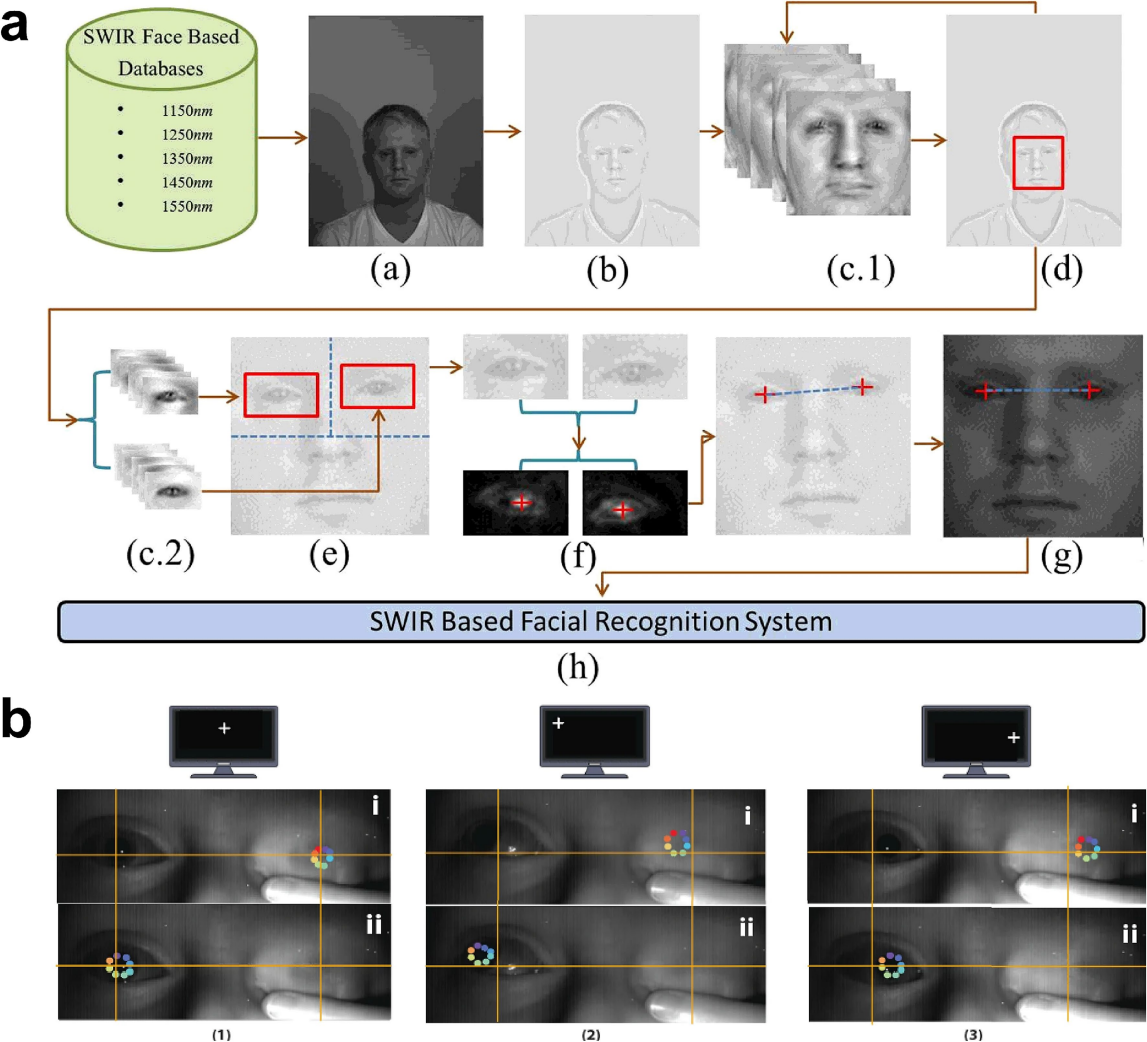
**(a)** Based on IR facial images (1,150 nm, 1,250 nm, 1,350 nm, 1,450 nm, and 1,550 nm), **(a)** outlines the proposed methodology for localizing pupil centers via summation range filtering, thereby facilitating face recognition through subsequent geometric normalization. Reproduced with permission from Whitelam and Bourlai (106). Copyright (2015) Elsevier. **(b)** Diagram of the DeepLabCut gaze tracking procedure. Reproduced from Ben Barak-Dror et al. (107) under the CC BY 4.0 license.

Earlier in the 1980s, Feldon et al. and Sollberger et al. reported the application of IR oculography in evaluating extraocular muscle function (102, 103). Utilizing IR eye-tracking technology, Matsui et al. designed the *Smart Eye Mask* device that enables non-invasive and portable sleep monitoring (104). Jiang et al. demonstrated that IR light enables precise stimulation of the posterior semicircular canal, which can elicit stable, reproducible, and direction-specific eye movements (105). This approach offers a safe, high-spatial-resolution strategy for non-invasive vestibular neuromodulation and the development of vestibular prostheses. In 2015, Whitelam et al. reported a novel method for achieving high-precision eye center localization in the IR band (1150–1,550 nm) (106). Figure 3a shows the proposed methodology for localizing pupil centers via summation range filtering based on IR facial images, thereby facilitating face recognition through subsequent geometric normalization. By employing a combination of two-dimensional normalized correlation coefficients and cumulative range filters, this approach accurately locates both eyes within a specific wavelength band. The research provides the foundation for geometric image normalization in facial recognition systems. Nir’s group reported a non-contact IR imaging technique capable of real-time monitoring of pupillary size and eye movement during closed-eye states (107). As shown in Figure 3b, during the experiment, the pupil position was tracked and marked with colored dots as participants directed their gaze to various locations on the screen. With eyes closed, horizontal and vertical eye movements toward the target direction were still clearly detected. The corresponding directional errors measured 8.9° in the vertical axis and 14° in the horizontal axis. Table 5 summaries the key parameters of recently reported IR eye tracking and pupillometry.

**Table 5:** Key parameters of recently reported IR eye tracking and pupillometry.

Disease	IR light	Sample size	Limitation	References
Eye localization	1,150–1,550 nm	135 subjects, 6,750 images	Frontal pose only, ideal lighting, limited dataset	Whitelam et al. (106)
Pupil center detection	850 nm	6 subjects, 7,938 images	Small dataset, no eyeglass wearers	Kondo et al. (50)
Visual fatigue	850 nm	20 subjects	Short viewing time, controlled lab environment	Kim et al. (96)
Eye-tracking	850 nm	/	Simulation only, no experimental fabrication	Zhao et al. (99)
Cognitive effort	760, 850 nm	32 subjects	Small sample, narrow age range, no difficulty levels	da Silva Soares et al. (95)
Pupillary light reflex, gaze estimation	1,100 nm	43 subjects	Head fixed, controlled dark room	Ben Barak-Dror et al. (107)

Compared with traditional eye tracking methods such as EOG and the search coil technique, IR video-oculography offers distinct advantages: EOG suffers from electrical artifacts and poor spatial resolution, while search coils are invasive and unsuitable for routine clinical use (96, 97). In contrast, IR oculography is non-contact, operates in darkness, and can track eye movements through closed eyelids (107), offering advantages for sleep monitoring, anesthesia, and vestibular assessment (104, 105). However, most IR eye tracking systems remain research prototypes (106, 107), and clinical adoption for ophthalmic diagnostics requires further validation.

## 6 IR based therapeutic applications in ophthalmology

When IR radiation approaches the resonant frequency of human cellular molecules, the significant thermal effects induced can increase dermal temperature, dilate capillaries, improve blood microcirculation, and enhance metabolic activity (108–110). Meanwhile, IR radiation can also influence various physiological processes through non-thermal mechanisms, such as stimulating nitric oxide synthesis, altering cell membrane potential, and modulating neurotransmitter release (42). IR lasers can be used to treat retinal diseases, adjust intraocular pressure elevation in glaucoma, reduce pinguecula, and treat macular edema (111, 112). Femtosecond lasers, which generate ultrafast pulsed lasers in the IR spectrum (1,053 nm), enable precise incisions during cataract surgery and corneal refractive surgery, enhancing surgical accuracy. Furthermore, integrating IR-sensitive photosensitizers enables the application of photodynamic therapy (31).

**Figure 4:**
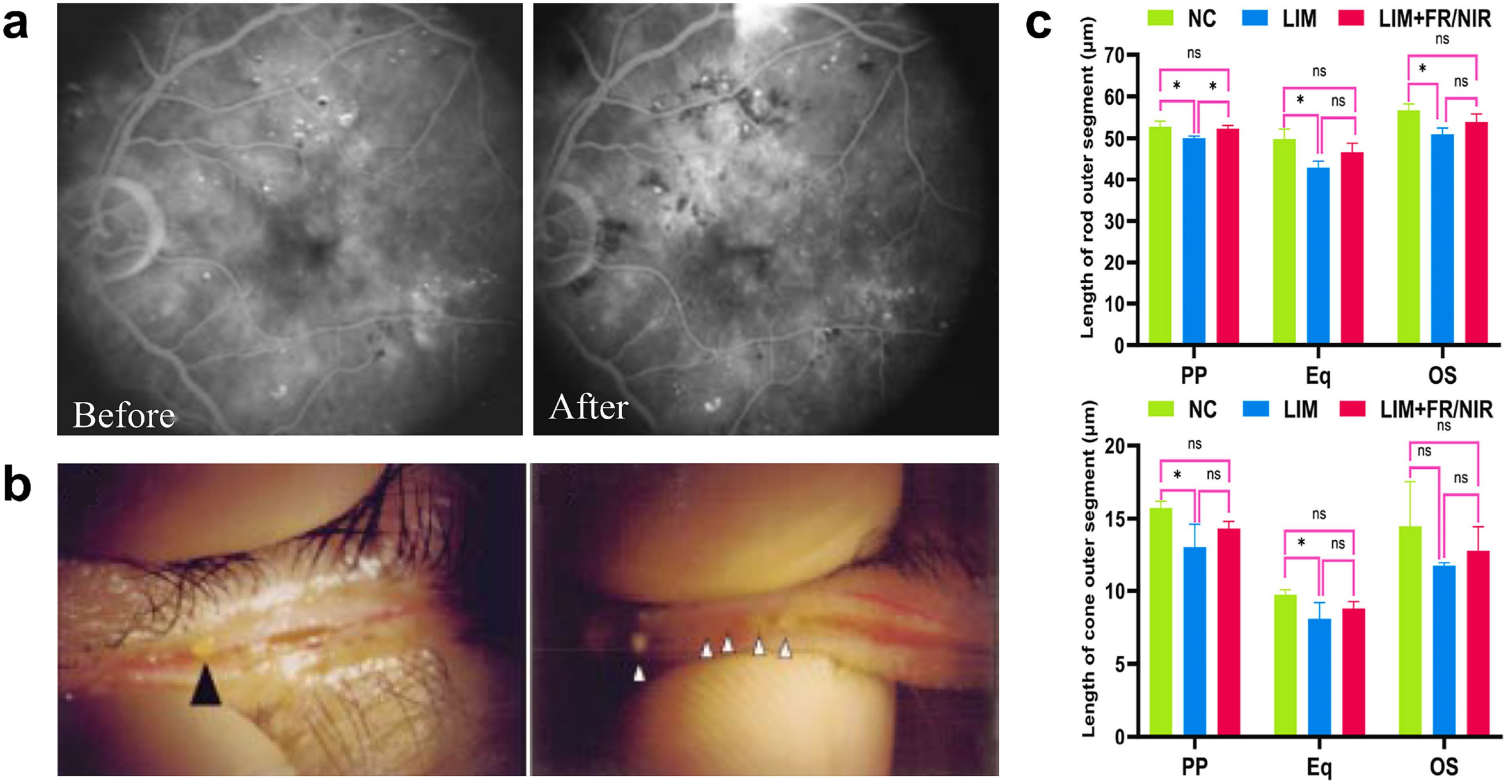
**(a)** Fluorescein angiography before and after laser treatment. Reproduced with permission from Friberg (113). Copyright (2001) Taylor & Francis. **(b)** Effect of IR warm compression on meibum secretion. Reproduced with permission from Goto et al. (114). Copyright (2002) BMJ Publishing Group. **(c)** Length of rod cell external segments and cone cell external segments in different retinal regions (PP: posterior pole; Eq: equator; OS: ora serrata) of three groups of guinea pigs. Reproduced with permission from Ding et al. (51). Copyright (2025) Elsevier.

In 2001, Friberg reported the therapeutic effects of 810 nm IR micro-pulse laser in diabetic macular edema (DME) (113). As shown in Figure 4a, diffuse leakage caused by DME is visible in the patient’s fundus. Subthreshold laser photocoagulation was subsequently applied to the superonasal quadrant of the macula. On follow-up angiography at 9 months, the laser-treated areas appeared as focal hypofluorescent spots, with complete resolution of exudates and minimal visibility of laser scars. In 2002, Goto et al. reported the efficacy and safety of infrared thermotherapy devices in treating non-inflammatory obstructive meibomian gland dysfunction (Figure 4b) (114). Prior to treatment, applying digital pressure revealed obstructed meibomian gland openings, making meibum expression nearly impossible, with an obstruction score of 3. After treatment, the device’s temperature helped to melt the solidified meibum, allowing for relatively easy expression with digital pressure, resulting in an obstruction score of 1. In 2006, Parodi et al. reported the effective treatment of macular edema secondary to branch retinal vein occlusion using subthreshold IR micro-pulse diode laser grid photocoagulation (115). Importantly, compared to conventional krypton laser threshold grid photocoagulation, this technique causes no visible laser-induced damage and demonstrates a superior safety profile. In 2025, Ding et al. systematically investigated the intervention effects of IR light on lens-induced myopic guinea pig models and the potential mechanisms (51). Figure 4c shows the length of rod cell external segments and cone cell external segments in different retinal regions (posterior pole, equatorial region, and sawtooth margin) of three groups of guinea pigs. NC, LIM, FR/IR represent the normal control group, lens-induced myopia group, and IR light group, respectively. The outer segment of photoreceptor cells plays a critical role in phototransduction. A decrease in outer segment length can compromise the efficiency of light signal capture, leading to impaired visual function. Induction of myopia is associated with structural damage to photoreceptor cells, which is often reflected by a reduction in cellular length. IR light intervention contributes positively to the restoration of photoreceptor cell length. Table 6 summaries the key parameters of recently reported IR based therapeutic applications in ophthalmology.

**Table 6:** Key parameters of recently reported IR based therapeutic applications in ophthalmology.

Disease	IR light	Sample size	Limitation	References
Choroidal melanoma	700–900 nm	Human: 7 eyes	Very small sample, short follow-up	Journée-De Korver et al. (108)
Nonexudative AMD	810 nm	229 eyes, 152 patients	CNV rate not significantly different	Olk et al. (111)
Nonexudative AMD	810 nm	100 eyes, 50 patients	No sham control, insufficient CNV events for analysis	Rodanant et al. (112)
Diabetic macular edema	810 nm	Threshold: 120 eyes; Subthreshold: 20 eyes	Underpowered, retrospective, short follow-up	Friberg et al. (113)
BRVO-related macular edema	810 nm	36 eyes (SGLT 17, TGLT 19)	Small sample, non-standard ETDRS protocol	Parodi et al. (115)
Dry eye	850–1,050 nm	37 patients	No control group, small sample, short follow-up	Goto et al. (114)
Dry eye	5–18 μm	61 eyes, 61 patients	No control group, objective markers not measured	Tian et al. (109)

## 7 Safety and limitations

To provide a clearer clinical perspective, we summarize the translational status of the key applications discussed in this review in Table 7. This classification is based on the level of evidence and the extent of integration into routine ophthalmic practice.

**Table 7:** Summary of clinical status of IR technology in ophthalmology.

Application	Technology	Clinical	Key evidence
Dry eye diagnosis	IR thermography	Research	Demonstrated correlation with symptoms, but lacks validation against standard tests (Schirmer, TBUT) in large cohorts (55, 59).
Meibography imaging	IR imaging	Clinical	Standard of care for morphological assessment of meibomian glands; integrated into commercial devices (Keratograph) (75).
Fundus imaging	IR imaging	Clinical	Established as a valuable adjunct in multimodal imaging for specific indications (69, 84, 85).
Eye tracking	IR video-oculography	Research	Used in specialized settings, particularly for vestibular function assessment and sleep monitoring (104, 105).
Therapeutic	IR micro-pulse Laser	Clinical	Established treatment for diabetic macular edema and macular edema secondary to retinal vein occlusion (113, 115).
Therapeutic	IR warm Compression	Clinical	Clinically available device for treating meibomian gland dysfunction (114).
Therapeutic	IR for myopia control	Research	Intervention effective in animal models; further validation and parameter standardization needed before human (51).
Therapeutic	Photodynamic Therapy	Research	Preclinical studies exploring IR sensitive photosensitizers for targeted therapy (31).

Though IR light-based technologies show significant prospects in ophthalmic diagnosis and treatment, their incorporation into routine clinical practice requires a comprehensive understanding of safety and practical limitations. Key factors that may influence clinical adoption include wavelength-dependent energy deposition, thermal risk, lack of standardized protocols, variable reproducibility, and evolving regulatory requirements (116–118).

Wavelength-dependent penetration: The penetration depth of IR radiation in ocular tissues is strongly wavelength-dependent, directly influencing clinical utility and safety (118). Near-IR light reaches the retina with low attenuation, enabling deep imaging (OCT, retinal oximetry). By contrast, mid- and far-IR wavelengths (>3 μm) are heavily absorbed by the tear film and corneal epithelium, confining penetration and thermal effects to the anterior segment (117). Exposure parameters must therefore account for this wavelength-specific energy deposition.

Ocular safety and thermal risks: Passive IR thermography operates well within safety limits, but active IR modalities must strictly comply with maximum permissible exposure (MPE) guidelines (ANSI Z80.36, ISO 15004-2, ICNIRP) (116, 118, 119). The lens and retinal pigment epithelium are particularly vulnerable to thermal damage: a retinal temperature rise of 4–10 °C can trigger photocoagulation, whereas sustained lenticular hyperthermia exceeding 3–5 °C may facilitate cataractogenesis (119, 120). Mitigation involves pulsed operation, active cooling, real-time temperature monitoring, and accounting for variations in pigmentation and lens status.

Standardization and reproducibility: Without standardized acquisition protocols, IR-based measurements lack cross-study comparability. Ambient temperature, acclimatization time, camera calibration, tear film stability, and region-of-interest definition all markedly influence reproducibility (117, 118).

Regulatory barriers: While some IR devices have received FDA clearance, many applications remain investigational (121). Regulatory approval for novel diagnostics demands costly, lengthy prospective trials that demonstrate safety, efficacy, and clinical utility (118, 122).

## 8 Conclusion and perspectives

This review systematically synthesizes recent advances in IR light-based technology applied to ophthalmology. Relative to conventional optical modalities, IR light-based techniques offer distinct advantages, including deeper tissue penetration, non-invasiveness, and enhanced diagnostic sensitivity. We highlight the fundamental principles underlying these technologies and their integration into diverse ophthalmic tools—encompassing IR thermography, IR imaging, functional monitoring, and IR based therapeutic applications. Importantly, we also critically examine safety considerations associated with IR exposure and identify the existing gaps between current research evidence and clinical practice. By addressing these challenges, this review aims to provide a balanced perspective on the translational pathway of IR technologies in ophthalmology.

Finally, we outline the key challenges and future prospects for IR light-based technology in ophthalmology: (1) Optimizing the resolution, sensitivity, and standardization of IR light based-technology to enable broader clinical application; (2) Standardizing treatment parameters, including determining the optimal irradiation wavelength, energy, and duration for various ocular diseases; (3) Developing compact, efficient, and cost-effective IR devices to overcome challenges in data interpretation and device portability, thereby enhancing clinical usability; (4) Achieving deep integration with artificial intelligence to analyze complex spectral data, significantly improving diagnostic automation and accuracy. With ongoing technological advancements, IR light-based technology holds promising potential for the early detection, monitoring of disease progression, and evaluation of treatments in ophthalmology.
